# Modification of NiO_*x*_ hole transport layer for acceleration of charge extraction in inverted perovskite solar cells†

**DOI:** 10.1039/d0ra00209g

**Published:** 2020-03-25

**Authors:** Zezhu Jin, Yanru Guo, Shuai Yuan, Jia-Shang Zhao, Xiao-Min Liang, Yujun Qin, Jian-Ping Zhang, Xi-Cheng Ai

**Affiliations:** Department of Chemistry, Renmin University of China Beijing 100872 China yjqin@ruc.edu.cn xcai@ruc.edu.cn

## Abstract

The modification of the inorganic hole transport layer has been an efficient method for optimizing the performance of inverted perovskite solar cells. In this work, we propose a facile modification of a compact NiO_*x*_ film with NiO_*x*_ nanoparticles and explore the effects on the charge carrier dynamic behaviors and photovoltaic performance of inverted perovskite devices. The modification of the NiO_*x*_ hole transport layer can not only enlarge the surface area and infiltration ability, but also adjust the valence band maximum to well match that of perovskite. The photoluminescence results confirm the acceleration of the charge separation and transport at the NiO_*x*_/perovskite interface. The corresponding device possesses better photovoltaic parameters than the device based on control NiO_*x*_ films. Moreover, the charge carrier transport/recombination dynamics are further systematically investigated by the measurements of time-resolved photoluminescence, transient photovoltage and transient photocurrent. Consequently, the results demonstrate that proper modification of NiO_*x*_ can significantly enlarge interface area and improve the hole extraction capacity, thus efficiently promoting charge separation and inhibiting charge recombination, which leads to the enhancement of the device performances.

## Abstract 2

The NiO_*x*_ layer modified with NiO_*x*_ nanoparticles obtains surface property optimization and energy level modulation, thus improving charge transport and device performance.

## Graphical abstract

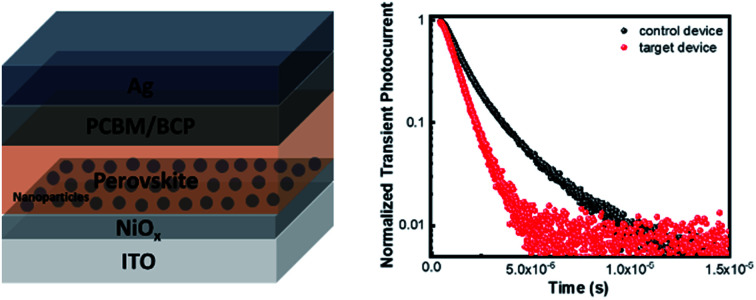

## 1. Introduction

During the past several years, organic–inorganic metal halide perovskite materials have attracted intensive research in the field of solar cells owing to their strong absorption capacity, excellent defect tolerance, long carrier diffusion length and high carrier mobility.^1,2^ The power conversion efficiency (PCE) record of perovskite solar cells (PSCs) has increased remarkably from 3.8% in 2009 to 25.2% recently.^3,4^ The traditional construction of PSCs could be divided into planar and mesoporous structures. In the planar-structured device, a compact TiO_2_ layer acts as the electron transport layer (ETL) to support the perovskite layer, while there is an additional mesoporous TiO_2_ (meso-TiO_2_) layer on the compact TiO_2_ in the mesoporous PSCs.^5,6^ The PSCs with meso-TiO_2_ layer derive from the dye-sensitized solar cells,^7^ in which the meso-TiO_2_ could provide the enlarged perovskite/ETL contact area and the photogenerated electrons can be injected from perovskite into ETL very quickly and sufficiently.^8,9^ In the subsequent development of PSCs, their structures have been varied from conventional configuration (n–i–p) to inverted configuration (p–i–n). Now all the perovskite devices of different structures have been widely explored and demonstrated high photovoltaic performances.^10,11^ Among these, the inverted PSCs have received increasing attention owing to their advantages of low processing temperature and negligible hysteresis effects.^12,13^ In inverted PSCs, hole transport layer (HTL) plays an important role in the photovoltaic performances. The appropriate hole transport materials (HTMs) can significantly optimize the Schottky contact, facilitate the interfacial charge separation and reduce the electron–hole recombination.

The organic HTMs inherited from conventional configuration have been first used in inverted PSCs owing to the mild fabrication process, while they are limited in the practical application to some extent. For examples, the widely used poly(3,4-ethylenedioxythiophene):poly(styrene-sulfonate) (PEDOT:PSS) and poly[*N*,*N*′-bis(4-butylphenyl)-*N*,*N*′-bis(phenyl)benzidine] (poly-TPD) suffer from the stability problem caused by the acidic and hygroscopic nature and the high production cost, respectively.^14,15^ Subsequently, the inorganic materials, including CuSCN,^16^ CuI^17^ and nickel oxide (NiO_*x*_),^18^ have been introduced as promising HTM candidates,^19^ which possess the advantages of low cost, high hole mobility and excellent chemical stability. Particularly, NiO_*x*_ is an attractive p-type semiconductor HTM with ease of synthesis, high light transmittance and energy level tunability.^20^ In addition, the doping or modification of NiO_*x*_ layer can further optimize the surface property, improve the conductivity and modulate the energy level, thereby contributing to the growth of high-quality perovskite films and improving the charge transport capability of HTL.^21,22^ Recently, inspired by the conventional meso-PSCs, the meso-structure has been adopted in the fabrication of the NiO_*x*_ HTL of inverted devices. For example, Zhang *et al.* reported the employment of Zn^2+^-doped CuGaO_2_ as HTM scaffold on compact NiO_*x*_ (c-NiO_*x*_) in the inverted PSCs, which obtained significant photovoltaic performance improvement due to the enlarged interfacial contact area and the increased HTL conductivity.^23^ Han *et al.* fabricated a hybrid HTL of meso-Al_2_O_3_/c-NiO_*x*_ and improved the photovoltaic performances of the inverted PSC owing to the minimized light absorption loss and interfacial recombination loss.^24^ Similarly, Alex *et al.* developed a meso-structure from Cu:NiO_*x*_ nanoparticle at c-NiO_*x*_/perovskite interface which significantly enhanced the hole mobility and the short-circuit current (*J*_SC_), as well as decreased series resistance of the HTL.^25^ Compared with the conventional inverted PSCs based-on NiO_*x*_ HTL, these works have proposed novel strategy for the modification of NiO_*x*_ with mesoporous layer and exhibited the advantages of interface optimization, carrier transport improvement and carrier recombination suppression.

In this work, we have developed a facile method to modify NiO_*x*_ HTL of the inverted PSCs with NiO_*x*_ nanoparticles (NPs). The increased surface roughness and infiltrating ability of modified NiO_*x*_ films is beneficial to the growth of high-quality perovskite films. The devices based on the modified NiO_*x*_ layer demonstrate an impressive photovoltaic behavior improvement owing to the enlarged interface area and the optimized matching of valence band maximum (VBM) of NiO_*x*_ and perovskite. The charge carrier transport/recombination dynamics of different perovskite films and devices are systematically investigated by the measurements of time-resolved photoluminescence (TRPL), transient photovoltage (TPV) and transient photocurrent (TPC), respectively. Consequently, the results reveal that the modification of NiO_*x*_ can significantly improve the hole extraction capacity and reduce the charge recombination behaviors, which results in the superior photovoltaic performance of the corresponding device.

## 2. Experimental

### 2.1 Fabrication of PSCs

The information of the reagents and chemicals used in this work are provided in the ESI.† Laser-ablated ITO glass was successively rinsed with detergent, deionized water, acetone and ethanol by an ultrasonic bath for 30 min, respectively. Subsequently, the substrate was treated by oxygen plasma for 20 min. For c-NiO_*x*_ HTL fabrication, NiO_*x*_ precursor solution (see solution preparation method in ESI†) was spin-coated on ITO substrate at 4000 rpm for 45 s, and then sintered at 300 °C for 1 h in air. The obtained c-NiO_*x*_ films were used for subsequent devices preparation and film characterization. For the c-NiO_*x*_ films modified with NiO_*x*_ NPs, denoted as m-NiO_*x*_, NiO_*x*_ NPs (see synthesis process in ESI†) were spin-coated on the c-NiO_*x*_ films at 3000 rpm for 30 s, then annealed at 100 °C for 20 min in air.

The perovskite active-layer was deposited by a processed method in the nitrogen-filled glovebox. The perovskite ([(FAPbI_3_)_0.85_(MAPbBr_3_)_0.15_]_0.95_(CsPbI_3_)_0.05_) (FA = formamidine, MA = methylamine) precursor solution was prepared by mixing FAI (1.0 M), PbI_2_ (1.1 M), MABr (0.2 M), PbBr_2_ (0.2 M) in anhydrous DMF : DMSO (DMF = *N*,*N*-dimethylformamide, DMSO = dimethyl sulfoxide, 4 : 1, v : v) mixed solvent, with a slight amount of excessive PbI_2_.^26^ After stirring for 1 h, 35 μL of 2.0 M CsI in DMSO was added to the mixed perovskite precursor to achieve the desired triple-cation composition. The solution was spin-coated on the substrates in a two-step program at 1000 and 5000 rpm for 10 and 20 s, respectively. During the second step, 200 μL of chlorobenzene (CB) was poured on the spinning substrate 5 s prior to the end of the program. Then the samples were annealed at 100 °C for 30 min. For PC61BM ([6,6]-phenyl-C61-butyric acid methylester) coating, its solution in CB (20 mg mL^−1^) was spin-coated at 1500 and 2000 rpm for 6 s and 40 s, respectively, and dried at 80 °C for 20 min. Finally, a 5 nm-thick bathocuproine and a 100 nm-thick Ag counter electrode were deposited by thermal evaporation. The final devices based on c-NiO_*x*_ and m-NiO_*x*_ are denoted as “c-PSCs” and “m-PSCs”, respectively.

The information of the instruments for the characterization is provided in ESI,† including scanning electron microscopy (SEM), transmission electron microscopy (TEM), atomic force microscopy (AFM), dynamic light scattering (DLS), contact angle (CA), X-ray diffraction (XRD), UV-vis absorption, steady-state photoluminescence (PL) spectroscopy, TRPL, ultraviolet photoelectron spectroscopy (UPS), current density (*J*)–voltage (*V*) characteristics and incident photon-to-current efficiency (IPCE) measurement.

### 2.2 Transient photoelectric experiments

TPV and TPC measurements were conducted following previous report.^27^ The targeted cell was kept under open-circuit condition and irradiated by a continuous-wave LED laser (520 nm, RGB photonics, Lambda beam) to maintain a steady-state photovoltage (*V*_ph_). Then weak laser pulses (532 nm, 7 ns) were applied to induce a small increase (Δ*V*_ph_) in *V*_ph_, with Δ*V*_ph_/*V*_ph_ ≤ 5%. Finally, electric signals were recorded by a digital oscilloscope (64 Xs, Lecroy; input impedance, 1 MΩ). A series of desired *V*_ph_ were obtained by adjusting the intensity of the LED laser through laser power and neutral filters. Corresponding TPC measurement was carried out by immediately switching the input impedance to 50 Ω after TPV measurement. The obtained photovoltage and photocurrent decay traces were fitted by exponential functions, and the charge recombination and transport lifetimes were weighted average values.

## 3. Results and discussion

The modification of c-NiO_*x*_ is realized through a facile method with the deposition of NiO_*x*_ NPs, which are obtained by a chemical precipitation method using Ni(NO_3_)_2_·6H_2_O as nickel source (see ESI† for preparation details).^28^ The as-prepared NiO_*x*_ NPs are visualized by TEM, as shown in Fig. S1,† which reveals the morphology of the particles aggregated to some extent. The NiO_*x*_ NPs are further measured with DLS to obtain the size distribution. As depicted in Fig. S2,† the particle size exhibits a relative narrow distribution with an average of about 20 nm. The NPs are also characterized with XRD and the pattern is shown in Fig. S3.† The diffraction peaks at 37.1°, 43.1° and 62.6° can be attributed to the (111), (200) and (220) crystal planes of cubic phase of NiO_*x*_.^29^ The transparency of the c-NiO_*x*_ and m-NiO_*x*_ on ITO glass substrate is measured and their optical transmittance spectra are shown in Fig. S4.† Obviously, the introduction of NiO_*x*_ NPs only leads to a little of transparency decrease and both the samples exhibit good light transmittance (>80%) in the visible region, which insures the light utilization rate of the perovskite layer.^30^

**Fig. 1:**
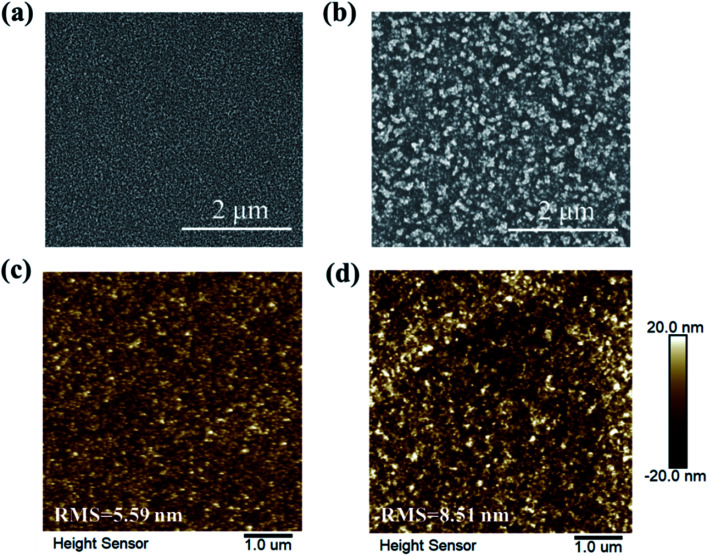
The SEM images of (a) c-NiO_*x*_ and (b) m-NiO_*x*_ HTLs. AFM topographic images of (c) c-NiO_*x*_ and (d) m-NiO_*x*_ HTLs.

SEM images of the two kinds of HTLs are observed and shown in Fig. 1. Apparently, the films both have excellent coverage, while compared with the smooth surface of c-NiO_*x*_ (Fig. 1a), the surface of m-NiO_*x*_ (Fig. 1b) looks much rougher owing to the homogeneously distributed NiO_*x*_ NPs. The roughness is expected to provide enlarged contact area with the subsequent perovskite layer. The surface topography is further examined by AFM, which reveals that the root-mean-square (RMS) value increases from 5.59 nm of c-NiO_*x*_ (Fig. 1c) to 8.51 nm of m-NiO_*x*_ (Fig. 1d) after the deposition of NiO_*x*_ NPs. The CA test is performed to investigate the infiltrating ability of the different HTLs against the perovskite precursor solution. As exhibited in Fig. S5,† the c-NiO_*x*_ and m-NiO_*x*_ substrates display CAs of 15.2° and 8.3°, respectively, indicating that the coating of NiO_*x*_ NPs layer can help the perovskite solution spread better. The changes of the surface properties of NiO_*x*_ film upon modification, as will be discussed later, would favor the growth of perovskite film and the optimization of NiO_*x*_/perovskite interface.

**Fig. 2:**
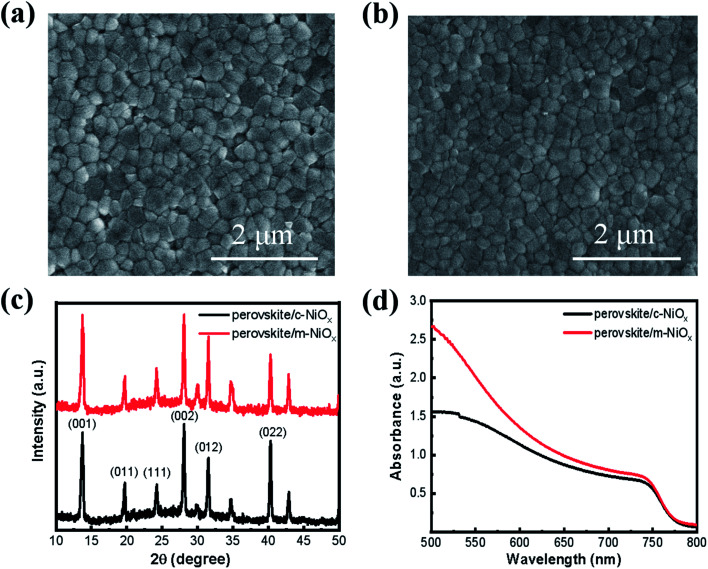
The SEM images of perovskite films based on (a) c-NiO_*x*_ and (b) m-NiO_*x*_ HTLs. (c) XRD patterns and (d) absorption spectra of perovskite films deposited on different HTLs.

The influence of the different NiO_*x*_ substrates on the morphology and crystal structures of perovskite films is characterized with SEM and XRD. As shown in Fig. 2, compared with the perovskite film on the c-NiO_*x*_ (Fig. 2a), the film on m-NiO_*x*_ presents more smooth and compact morphology (Fig. 2b), which indicates that the modification of substrates could influence the growth of perovskite film. The statistic distributions from the SEM images also displays that the perovskite grains on m-NiO_*x*_ possess larger average size (245 nm) than those on c-NiO_*x*_ (230 nm) (Fig. S6†), as well as narrower size distribution. The enlarged perovskite grains with reduced grain boundary can effectively decrease defect density, which would benefit the device performance. XRD patterns of the perovskite films on different NiO_*x*_ films are illustrated in Fig. 2c, which reveals the typical perovskite characteristics of α black phase rather than δ non-perovskite yellow phase.^31^ The strong bragg peaks at 13.74°, 19.7°, 24.2°, 28.08°, 31.52° and 40.31° are assigned to the (001), (011), (111), (002), (012) and (022) perovskite crystal planes, respectively, indicating NiO_*x*_ NPs has no obvious influence on the crystal structures of perovskite under the same preparation conditions of perovskite. In addition, no XRD peaks of residual PbI_2_ were found in the two samples.^32^ The UV-vis absorption of the perovskite films on c-NiO_*x*_ and m-NiO_*x*_ is also investigated, as shown in Fig. 2d. The improved crystallinity of perovskite on m-NiO_*x*_ is beneficial to the photon absorption, leading to the gradual rise of the absorption from 750 nm. The absorption ability enhancement of perovskite/m-NiO_*x*_ would improve *J*_SC_ of the corresponding device.

The Fermi energy (*E*_F_) levels of the NiO_*x*_ films are measured to investigate the influence of NiO_*x*_ NPs modification. Generally, the *E*_F_ value of NiO_*x*_ varies between −4.7 eV and −5.5 eV, depending on its preparation process.^33^ According to the UPS curves (Fig. S7†), the *E*_F_ values are −4.79 eV and −4.95 eV for c-NiO_*x*_ and m-NiO_*x*_, respectively, and the VBM of m-NiO_*x*_ is −5.36 eV, which is slightly lower than −5.22 eV of c-NiO_*x*_ (see ESI† for calculation details). The value of m-NiO_*x*_ is much closer to the VBM of the perovskite (−5.60 to −5.40 eV),^34–36^ which can contribute to the improvement of charge collection and photovoltaic parameters.

**Fig. 3:**
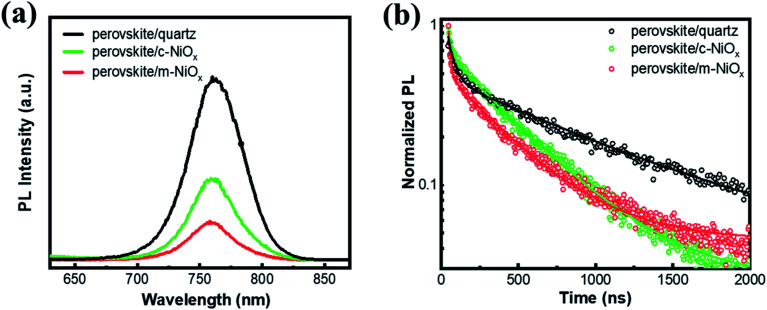
(a) Steady-state PL spectra and (b) PL decay dynamics of perovskite films deposited on different substrates measured at 760 nm. The hollow circles represent experimental data and the solid lines are the fitting results in panel (b).

To investigate the photogenerated carrier extraction abilities of different HTLs from the perovskite absorber, the steady-state PL spectra and PL decay dynamics are measured, with the perovskite films on quartz as reference. As seen in Fig. 3a, all the perovskite films exhibit the luminescence peaks at about 765 nm, consistent with the band-edge absorption from the UV-vis absorption spectra. Compared with that of perovskite/quartz, the PL emission of perovskite/HTL samples is dramatically quenched owing to the efficient carrier extraction of NiO_*x*_. Moreover, the m-NiO_*x*_ shows a considerably greater PL quenching ability than c-NiO_*x*_, implying the promotion of the charge collection and transport efficiency of the former. In details, m-NiO_*x*_ possesses superior hole extracting ability from the perovskite, which efficiently restricts the PL emission owing to the less recombination of photogenerated electrons and holes in perovskite. The excellent PL quenching ability of m-NiO_*x*_ results from the enlarged contact area at the perovskite/m-NiO_*x*_ interface and the optimized energy level alignment with perovskite. As mentioned above, the hole transport from perovskite to m-NiO_*x*_ is much preferable than to c-NiO_*x*_ owing to the better matched VBMs of m-NiO_*x*_ and perovskite. This result is also in agreement with the transient photoelectric experiments (to be discussed in Fig. 5).

To further explore the behaviors of the charge carriers in the three perovskite films, the TRPL experiment is conducted and the results are presented in Fig. 3b. The TRPL data are fitted with the biexponential function as below:
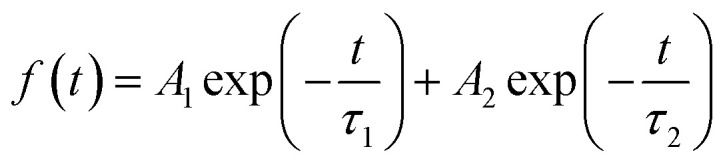
where *A*_1_ and *A*_2_ are pre-exponential factors and *τ*_1_ and *τ*_2_ are time constants. The detailed fitting parameters are summarized in Table 1. All the samples display biexponential decay behavior with fast and slow regions. For the perovskite/quartz sample, the transient PL decay behavior exhibits a long carrier lifetime. The fast lifetime *τ*_1_ attributed to the band-to-band recombination is 40.63 ns, while the slow lifetime *τ*_2_ is 943.13 ns, which is closely related to the trap-assisted recombination.^37,38^ For the perovskite/HTL samples, *τ*_1_ is attributed to charge extraction process from perovskite to HTL. The perovskite/c-NiO_*x*_ sample gives a *τ*_1_ value of 22.65 ns while the *τ*_1_ value of perovskite/m-NiO_*x*_ decreases to 13.60 ns, suggesting better charge extraction capability owing to the modification of the NiO_*x*_ film. In addition, the proportion of fast component (*A*_1_) in the perovskite/m-NiO_*x*_ sample is larger than that in the perovskite/c-NiO_*x*_ sample, indicating that charge extraction from perovskite to m-NiO_*x*_ is more dominant in the perovskite/m-NiO_*x*_ sample. As for *τ*_2_ related to trap-assisted recombination, the lower value of perovskite/m-NiO_*x*_ (385.59 ns) than perovskite/c-NiO_*x*_ (421.58 ns) indicates that the perovskite film on m-NiO_*x*_ has less trap state density. In addition, the smaller *A*_2_ value of perovskite/m-NiO_*x*_ compared with perovskite/c-NiO_*x*_ suggests the later coming of the corresponding slow component, in other words, the later involvement of the trap-assisted recombination process.

**Table 1:** Fitting parameters extracted from the PL decay traces

Sample	*A*_1_	*τ*_1_	*A*_2_	*τ*_2_
Perovskite/quartz	0.51	40.63	0.49	943.13
Perovskite/c-NiO_*x*_	0.33	22.65	0.67	421.58
Perovskite/m-NiO_*x*_	0.53	13.60	0.47	385.59

**Fig. 4:**
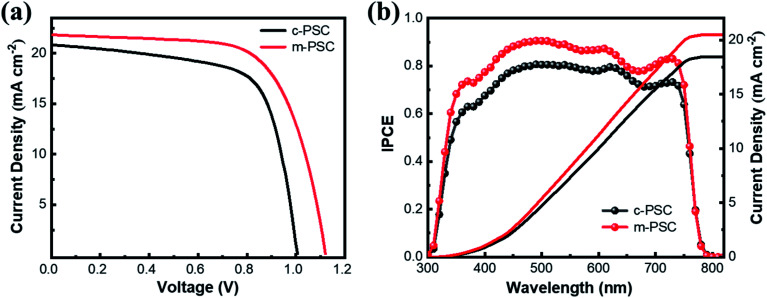
(a) *J*–*V* curves and (b) IPCE curves with the corresponding integrated photocurrent of the different HTLs based PSCs.

The devices based on the two kinds of NiO_*x*_ as HTLs are fabricated and their photovoltaic performance are compared (the devices based on c-NiO_*x*_ and m-NiO_*x*_ are denoted as c-PSCs and m-PSCs, respectively). The cross-sectional SEM images of the two devices are shown in Fig. S8.† Each image clearly demonstrates the distinct layers of the corresponding PSC. Obviously, the HTLs of the device exhibit different thickness with ∼23 nm for c-NiO_*x*_ and ∼35 nm m-NiO_*x*_ owing to the modification of the latter with NiO_*x*_ NPs. It is notable that the thickness of perovskite layer of m-PSC (460 nm) is a little larger than that of c-PSC (440 nm), which could be attributed to the better wettability of m-NiO_*x*_ for favorable perovskite deposition. The statistical distribution of the photovoltaic parameters of the PSCs is presented in Fig. S9,† which exhibits the superiority of m-PSCs. The *J*–*V* curves of the best c-PSC and m-PSC are displayed in Fig. 4a. The c-PSC exhibits PCE of 14.2% with open-circuit voltage (*V*_OC_) of 1.01 V, *J*_SC_ of 20.8 mA cm^−2^ and fill factor (FF) of 0.67, while the m-PSC demonstrates enhanced performance with PCE of 16.4%, *V*_OC_ of 1.12 V, *J*_SC_ of 21.8 mA cm^−2^ and FF of 0.67. Obviously, the improved PCE of m-PSC is primarily attributable to the increasing *J*_SC_ and *V*_OC_. The increase of *J*_SC_ of m-PSCs could be ascribed to the enhancement of light absorbance, consistent with the UV-vis absorption (Fig. 2d). The increase of *V*_OC_ of m-PSCs is mainly due to the reduction of energy loss with m-NiO_*x*_ as HTL. As mentioned, compared with c-NiO_*x*_, the better wettability and rougher surface of m-NiO_*x*_ could favor the perovskite growth and optimize the perovskite/HTL interface contact, which facilitates the charge transport and reduces the charge recombination. Moreover, the better VBM alignment of m-NiO_*x*_ with perovskite than that of c-NiO_*x*_ also benefits the charge extraction from perovskite and injection to HTL. Fig. 4b shows the IPCE spectra for the devices with the corresponding integrated photocurrent densities. The IPCE value correlated to m-PSC exceeds 80%, much higher than that of c-PSC. The higher IPCE means that the m-PSCs has a better utilization of incident light, thus leading to a superior PCE. Meanwhile, the integrated current density is 20.44 and 18.42 mA cm^−2^ for the two devices, corresponding to the *J*_SC_ value obtained from the statistical parameters (Fig. S9a†). Considering the similar fabrication conditions, the great improvement in photovoltaic performance of m-PSCs should be ascribed to enlarged charge separation area at the perovskite/HTL interface and the regulation of the energy level of m-NiO_*x*_ for enhanced charge transport and reduced charge recombination, which is to be proved in the following TPV and TPC results.

**Fig. 5:**
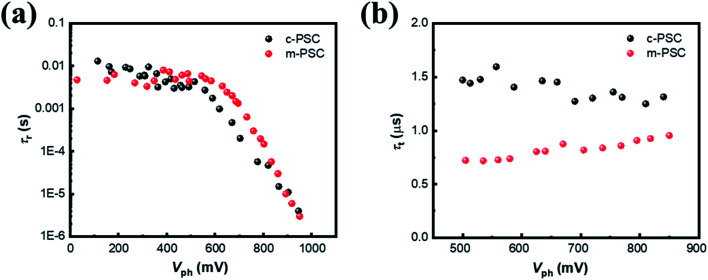
(a) The *τ*_r_–*V*_ph_ dependencies from TPV data for different PSCs. (b) The *τ*_t_–*V*_ph_ dependencies from TPC data at high voltage (>500 mV) for different PSCs.

To probe the origin for photoelectric performance improvement and further unravel the underlying carrier behaviors in the devices, TPV is performed to study the charge recombination dynamics of the targeted PSCs.^39,40^ Fig. S10† illustrate the corresponding semi-logarithmic plots of normalized *V*_ph_ decay curves against time for c-PSC and m-PSC, respectively. Apparently, the two devices present distinct decay profiles. Charge recombination lifetimes (*τ*_r_) are obtained by exponential fitting of the decay traces (see ESI† for fitting details) and Fig. 5a shows the dependence of *τ*_r_ upon different *V*_ph_ for the two devices. From a rough view, the *τ*_r_ evolution tendency could be divided into two regions with a demarcation point around 550 mV. The *τ*_r_ values are almost constant at 7–8 ms in low voltage region, then *τ*_r_ drops sharply to several μs when the voltage increases from 550 mV to 1000 mV. The two-developing stage of *τ*_r_ along with *V*_ph_ could be assigned to different competitive recombination processes. In low voltage region, the photogenerated electrons are predominately distributed in deep trap states and the only pathway for a trapped electron/hole to meet or capture another hole/electron is hopping.^41^ For the two devices with the similar perovskite layer, the effect of NiO_*x*_ modification on charge separation and transport is negligible in this case,^42^ thus the almost similar recombination lifetime constants are observed. As the voltage increases continuously, the *E*_F_ occupied by holes gradually rises and approaches the valence band. Because the population behaviors of electrons and holes are similar, only the behavior of holes is discussed for simplification.^43^ In this case, it is easy for a trapped hole to accomplish interfacial charge separation driven by the extraction force at perovskite/HTL interface.^44,45^ The linear correlation between *τ*_r_ and *V*_ph_ at the semi-logarithmic scale is also observed, which suggests that the charge recombination process should follow the multiple-trap model.^46^ Compared with c-PSCs, m-PSCs have a longer *τ*_r_, indicating that the m-NiO_*x*_/perovskite interface could promote the charge separation and suppress the charge recombination efficiently. This is mainly attributed to the well matched VBMs of m-NiO_*x*_ and perovskite, as well as their increased interface contact area.

Subsequently, TPC measurement is conducted to investigate the charge carrier transport dynamics. Fig. S11† illustrate the corresponding semi-logarithmic plots of normalized photocurrent decay curves against time for different PSCs. Apparently, the devices present similar decay profiles under different *V*_ph_. The decay curves are fitted to get the charge transport lifetime (*τ*_t_) at different voltages with monoexponential decay function as depicted in ESI.† The dependency of *τ*_t_ on *V*_ph_ from 500 mV to 850 mV obviously exhibits a relatively constant tendency for both devices (Fig. 5b), similar to the previous reports.^47,48^ The *τ*_t_ values of m-PSC and c-PSC are 0.75 μs and 1.5 μs, respectively, indicating the more efficient charge transport for the former. The results of transient photoelectric experiments quantitatively reveal that the modified HTL behaves superiorly in suppressing charge recombination and elevating charge collection, which accounts for the improvement of the device photovoltaic performance.

## 4. Conclusions

In summary, a facile method to modify NiO_*x*_ HTL is proposed by additional deposition of NiO_*x*_ NPs in inverted planar PSCs. The modification can increase the HTL surface area and infiltrating ability, as well as modulate energy level of NiO_*x*_, which is beneficial to the growth of perovskite films and the acceleration of the charge separation and transport. TRPL results exhibits the enhanced charge extraction capability of NiO_*x*_ HTL owing to the modification. The transient photoelectric tests further reveal that the modification of NiO_*x*_ can efficiently promote charge transport/separation and reduce the charge recombination, which results in the superior photovoltaic performance of the device. This work could provide new strategies for developing high-efficiency perovskite devices and understanding the underlying charge carrier dynamics.

## Conflicts of interest

There are no conflicts of interest to declare.

## Supplementary Material

RA-010-D0RA00209G-s001
