# Supplementary material for: Modification of NiOx hole transport layer for acceleration of charge extraction in inverted perovskite solar cells
Source: RSC Adv. 2020 Mar 25;10(21):12289–96. doi: 10.1039/d0ra00209g (PMC9050867; doi:10.1039/d0ra00209g)
Supplement: RA-010-D0RA00209G-s001 [file RA-010-D0RA00209G-s001.pdf]

Electronic Supplementary Material (ESI) for RSC Advances.
This journal is © The Royal Society of Chemistry 2020

Supplementary Materials

Modification of NiO_x hole transport layer for acceleration of charge extraction in inverted perovskite solar cells

Zezhu Jin, Yanru Guo, Shuai Yuan, Jia-Shang Zhao, Xiao-Min Liang, Yujun Qin*, Jian-Ping Zhang and Xi-Cheng Ai*

Department of Chemistry, Renmin University of China, Beijing 100872, China.

S1. Experimental

S1.1. Materials

All the reagents and chemicals were used as received. Nickel nitrate hexahydrate ($Ni(NO_3)_2 \cdot 6H_2O$, 99.99%) was purchased from Aladdin. Formamidinium iodide (FAI, ⩾99.5%), methylammonium bromide (MABr, ⩾99.5%), [6,6]-phenyl C61 butyric acid methyl ester (PC61BM, ⩾ 99.5%) and 2,9-dimethyl-4,7-diphenyl-1,10-phenanthroline (bathocuproine, BCP) were purchased from Xi'an Polymer Light Technology Corp. Lead (II) iodide (PbI_2, ⩾ 99.999%), cesium iodide (CsI, ⩾ 99.998%), lead (II) bromide (MABr, ⩾ 99.999%), ethylene glycol and ethylenediamine were purchased from Alfa Aesar. N, N-dimethylformamide (DMF), dimethyl sulfoxide (DMSO) and chlorobenzene (CB) were purchased from J&K. All solvents were super dry with molecular sieves.

S1.2. Non-stoichiometric NiO_x NPs synthesis

NiO_x NPs were prepared by a chemical precipitation method according to the previous reports.[1] Briefly, to prepare the pristine NiO_x NPs, $Ni(NO_3)_2 \cdot 6H_2O$ (0.1 mol) was dispersed in 20 mL of deionized water to obtain a green solution under continuous magnetic stirring. But after a while, the solution was adjusted to pH = 10 by adding the NaOH solution (10 mol L^{-1}) dropwise. After continuous stirring for 10 min, the green colloidal precipitation was thoroughly washed with deionized water and dried at 80 °C for 6 h. The obtained green powder was then calcined at 270 °C for 2 h to obtain the

NiO_x NPs. The NiO_x NPs inks were dispersed in deionized water at a certain concentration.

S1.3. NiO_x precursor solution

The compact NiO_x films were prepared according to a hydrogel process. Briefly, 0.005 mol of $Ni(NO_3)_2 \cdot 6H_2O$ was dissolved in 10 mL of ethylene glycol with 350 μL ethylenediamine. The solution was stirred 12 h in air at room temperature and then the homogeneous dark blue solution was obtained.

S1.4. Characterization

The morphologies of the perovskite films and the cross-sectional SEM images of devices were characterized by field-emission scanning electron microscopy (FE-SEM, Hitachi SU8010) at an accelerating voltage of 5 kV. The roughness of different HTLs were characterized by atomic force microscopy (AFM, Dimension ICON, Bruker). The morphology of the NiO_x NPs was determined at 120 kV using transmission electron microscopy (TEM, JEOL JEM-2010). The particle size of NiO_x NPs was measured by dynamic light scattering (DLS) on the Zetasizer Nano ZS90 particle size and zeta potential analyzer (Malvern Instruments). The interfacial behavior was recorded by an optical contact angle (CA) measuring device (KRÜSS drop shape analyzer DSA30). X-ray diffraction (XRD) analysis was conducted on a Shimadzu XRD-7000 (power) using Cu Kα radiation at a scan rate of 2° per min in the 2θ range from 30° to 80° for NiO_x NPs and from 10° to 50° for perovskite/NiO_x samples. UV-vis absorption measurement was performed on a Cary 50 spectrometer. The steady-state photoluminescence (PL) spectroscopy was carried out by using an Edinburgh FLS980 spectrometer at an excitation wavelength of 510 nm with irradiation from the side of spin-coated materials. TRPL spectra was performed by a picosecond diode laser (EPL-470, 95 ps) and the signal was recorded at 760 nm after exciting at 510 nm. The ultraviolet photoelectron spectroscopy (UPS) was measured by Thermo Scientific ESCALab 250 Xi. The gas discharge lamp was used for UPS, with helium gas admitted and the He I (21.22 eV) emission line employed. The helium pressure in the analysis chamber during analysis was about 3E-8 mbar. The data was acquired with -10 V bias.

The current density (J)-voltage (V) characteristics were obtained by exposing the sample to a standardized sunlight simulator, which was calibrated by a standard silicon reference cell to AM 1.5G one-sun illumination (100 mW cm^{-2}), with sweeping over the predetermined applied bias voltage range from open circuit to short circuit at a scan rate of 10 mV s^{-1} without light soaking or voltage treatment history. A metal aperture mask was used to fix the active area to 0.1 cm^2, and all data was acquired by a Keithley 2400 Sourcemeter. The incident photon-to-current efficiency (IPCE) measurement was carried out with the photoelectric conversion test system (SCS100-X150-DSSC, Zolix Instruments Co., Ltd).

S2. Results and discussion

S2.1. Characterization of NiO_x NPs

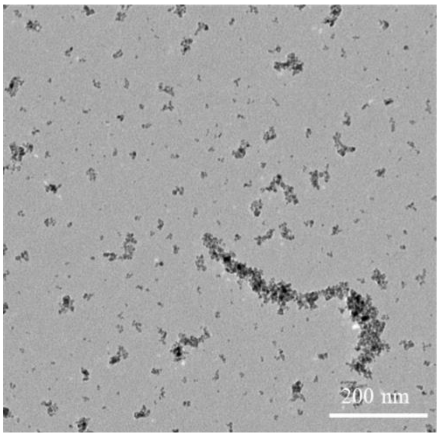

Fig. S1 TEM image of the NiO_x NPs.

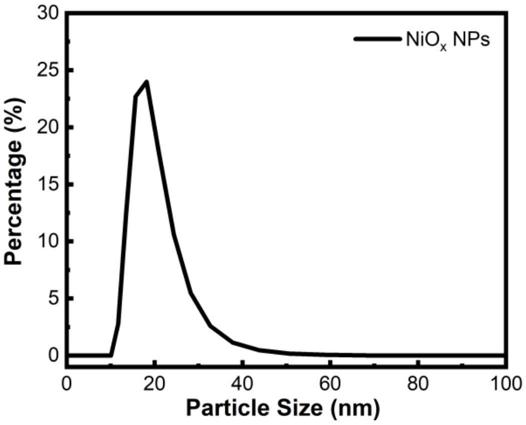

Fig. S2 DLS analysis result of the NiO_x NPs size distribution.

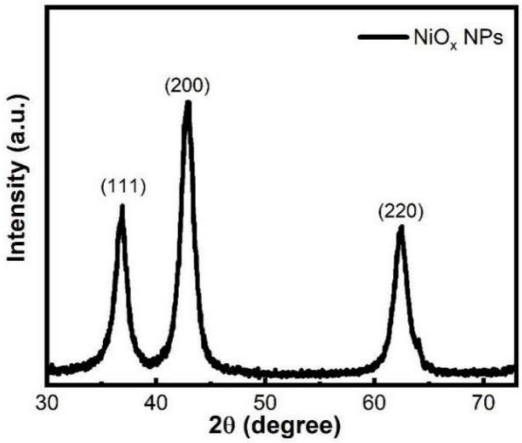

Fig. S3 XRD pattern of pure NiO$_x$ NPs.

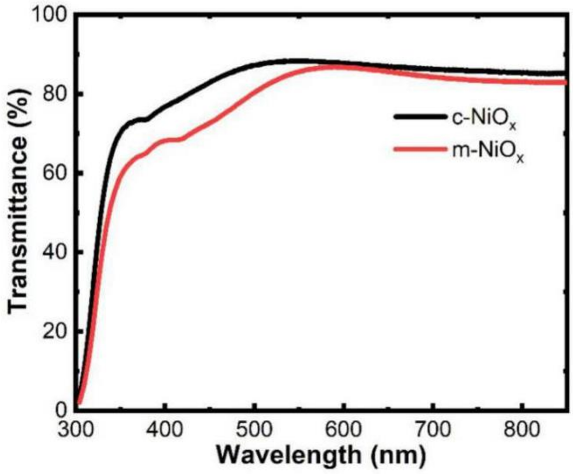

Fig. S4 Transmission spectra of NiO$_x$ on ITO glass substrate.

S2.2. Characterization of different NiO$_x$ HTLs

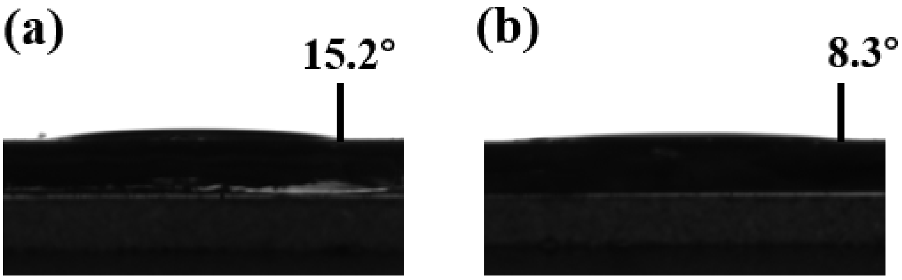

Fig. S5 Contact angle measurement of perovskite precursor solution (1.2 M) on (a) c-NiO$_x$ and (b) m-NiO$_x$ HTLs.

S2.3. The statistic distributions of the perovskite films

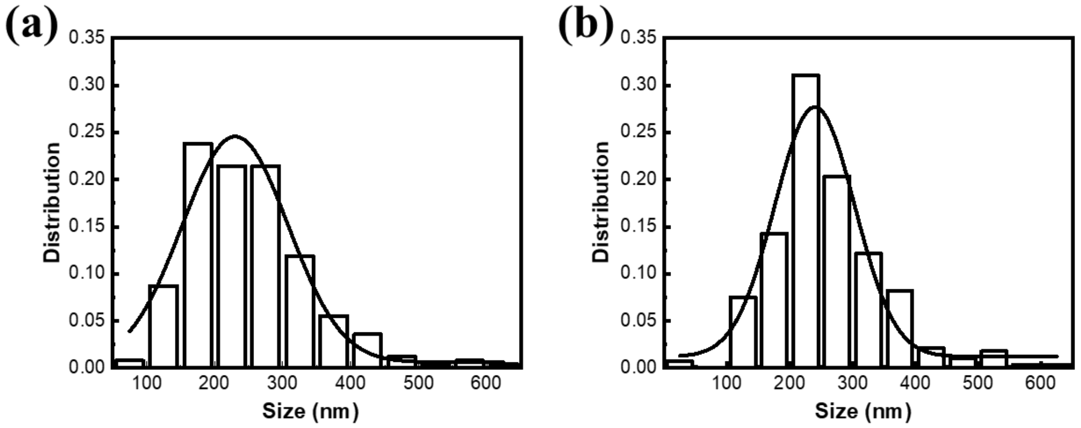

Fig. S6 The statistic distributions of the perovskite grain size from SEM images on (a) c-NiO_x and (b) m-NiO_x.

S2.4. Characterization of electronic structures of different NiO_x HTLs

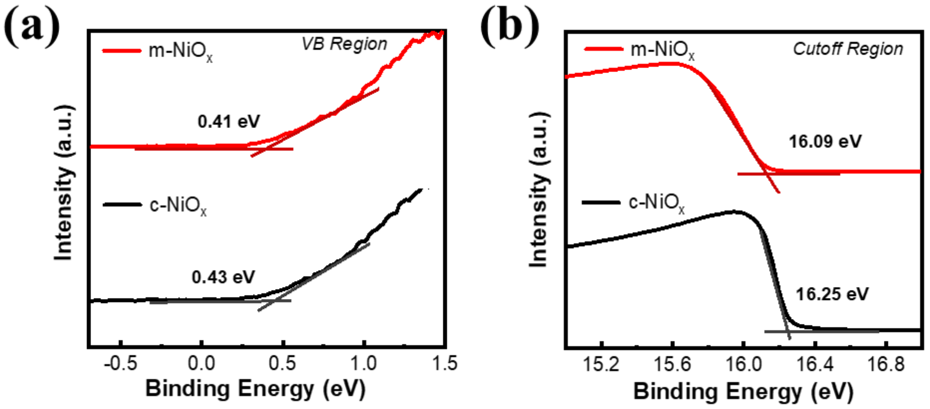

Fig. S7 UPS spectra of the different NiO_x HTLs (a) at VB region of low binding energy and (b) at cutoff region of high binding energy.

The E_F and VBM values of the NiO_x films are obtained from the UPS measurements. The UPS spectra of the different NiO_x films are shown in Fig. S7, with the curves corresponding to the valence band (VB) region at low binding energy (Fig. S7a) and the cutoff region at high binding energy (Fig. S7b). E_F is located in the band gap, and there is an energy difference (ΔE) between E_F and VBM, VBM = $E_F + \Delta E$.[2] ΔE could be determined through the intersect of the baseline and the linear extrapolation along the steep ascending part of the VB region. As exhibited in Fig. S7a, the ΔE values are 0.43 eV and 0.41 eV for c-NiO_x and m-NiO_x, respectively.

The E_F values are obtained from the UPS curves of Fig. S7b with $E_F = h\nu - (E_{F(Au)} - E_{cutoff})$. $h\nu$ = 21.22 eV upon the He I emission source. $E_{F(Au)}$ (21.4 eV) is E_F of Au standard sample connected with the tested NiO_x/ITO. E_{cutoff} is corresponding to the kinetic energy at the secondary cutoff edge (low kinetic cutoff edge). From Fig. S7b, the binding energy (E_B) values of cutoff edge are 16.25 eV and 16.09 eV for c-NiO_x and m-NiO_x, respectively. From $E_{cutoff} = h\nu - E_B$, the E_{cutoff} values are 4.97 eV and 5.13 eV for c-NiO_x and m-NiO_x, respectively. Thus, the E_F values are 4.79 eV and 4.95 eV for c-NiO_x and m-NiO_x, respectively.

Finally, with the equation VBM = $E_F + \Delta E$, the VBM values are -5.22 eV and -5.36 eV for c-NiO_x and m-NiO_x, respectively.

S2.5. Typical device structures and performance parameters

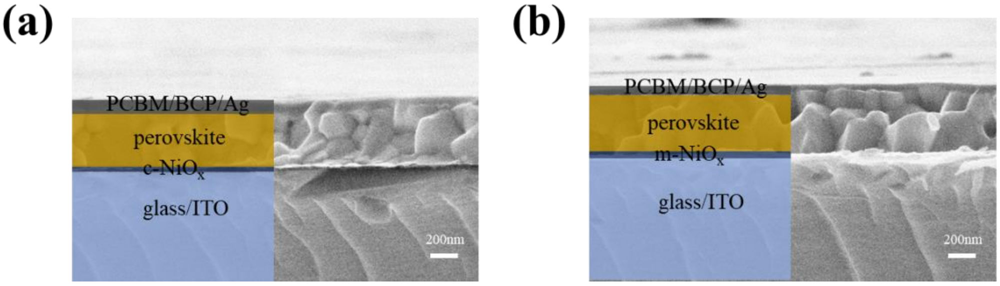

Fig. S8 Cross-sectional SEM images of (a) glass/ITO/c-NiO_x/perovskite/PCBM/BCP/Ag and (b) glass/ITO/m-NiO_x/perovskite/PCBM/BCP/Ag.

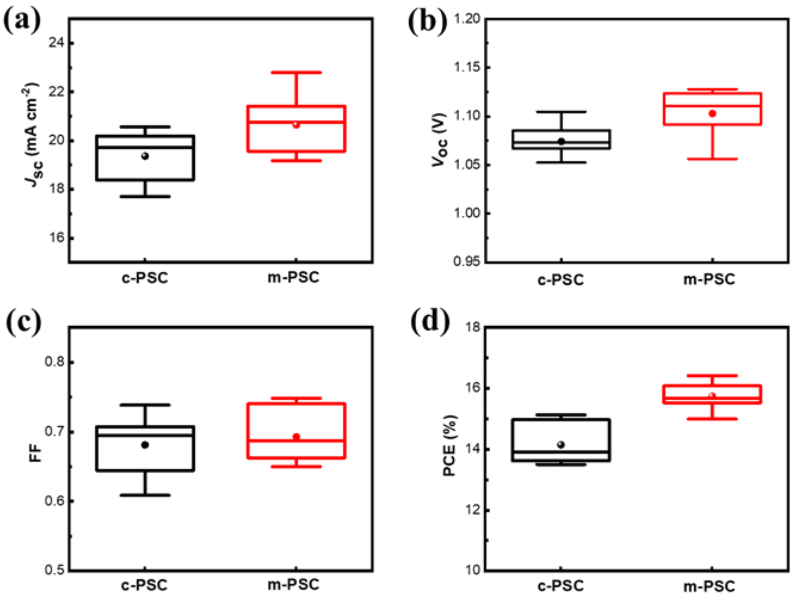

Fig. S9 Statistical parameters of (a) J_{SC}, (b) V_{OC}, (c) FF and (d) PCE for different devices under forward scan. Statistical measurements of fifteen samples were performed for the two devices, respectively.

S2.6. The fitting of TPV and TPC carrier dynamics analysis

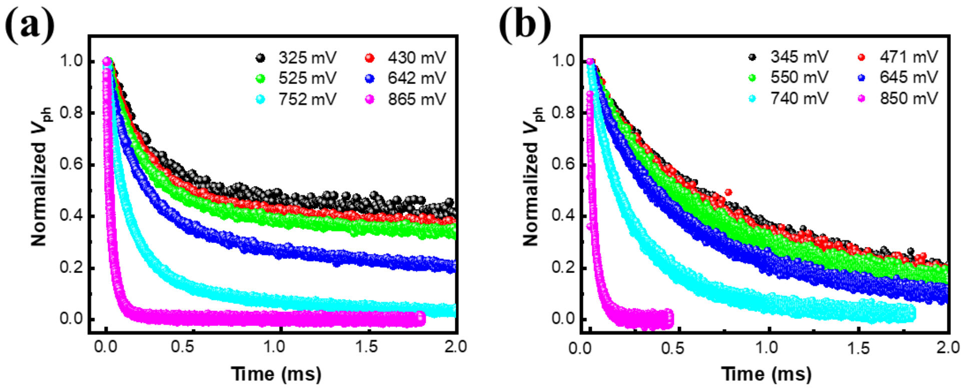

Fig. S10 Normalized TPV curves of (a) c-PSC and (b) m-PSC.

The raw TPV data are fitted with bi-exponential decay equation,

$$V_{\mathrm{ph}}(t) = V_{\mathrm{base}} + A_1 \exp\left(-\frac{t}{\tau_1}\right) + A_2 \exp\left(-\frac{t}{\tau_2}\right)$$

where V_{base} is the baseline, A_1 and A_2 are pre-exponential factors, and τ_1 and τ_2 are time constants. The average charge recombination time constants (τ_{r}) are calculated as shown below,

$$\tau_{\mathrm{r}} = \frac{A_1\tau_1 + A_2\tau_2}{A_1 + A_2}$$

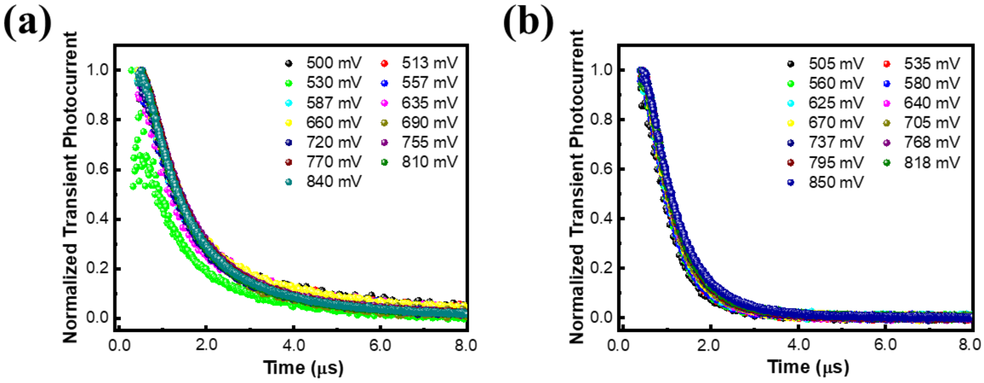

Fig. S11 Normalized TPC curves of (a) c-PSC and (b) m-PSC at high voltage (>500 mV).

The raw TPC data are fitted with monoexponential decay equation,

$$I_{\mathrm{ph}}(t) = A\exp\left(-\frac{t}{\tau_{\mathrm{t}}}\right)$$

where A is pre-exponential factor, and τ_{t} is charge transport time constant.

References

1 H. Zhang, J. Cheng, F. Lin, H. He, J. Mao, K. S. Wong, A. K. Jen and W. C. Choy, *ACS Nano*, 2016, **10**, 1503-1511.

2 W. Chen, Y. Zhou, L. Wang, Y. Wu, B. Tu, B. Yu, F. Liu, H. W. Tam, G. Wang, A. B. Djurisic, L. Huang and Z. He, *Adv. Mater.*, 2018, **30**, 1800515.
